# Population Pharmacokinetic Analyses for Omadacycline Using Phase 1 and 3 Data

**DOI:** 10.1128/AAC.02263-19

**Published:** 2020-06-23

**Authors:** Elizabeth A. Lakota, Scott A. Van Wart, Michael Trang, Evan Tzanis, Sujata M. Bhavnani, M. Courtney Safir, Lawrence Friedrich, Judith N. Steenbergen, Paul G. Ambrose, Christopher M. Rubino

**Affiliations:** a Institute for Clinical Pharmacodynamics, Inc., Schenectady, New York, USA; b Paratek Pharmaceuticals, King of Prussia, Pennsylvania, USA

**Keywords:** omadacycline, patients, population pharmacokinetics

## Abstract

Omadacycline, a novel aminomethylcycline antibiotic with activity against Gram-positive and -negative organisms, including tetracycline-resistant pathogens, received FDA approval in October 2018 for the treatment of patients with acute bacterial skin and skin structure infections (ABSSSI) and community-acquired bacterial pneumonia (CABP). A previously developed population pharmacokinetic (PK) model based on phase 1 intravenous and oral PK data was refined using data from infected patients. Data from 10 phase 1 studies used to develop the previous model were pooled with data from three additional phase 1 studies, a phase 1b uncomplicated urinary tract infection study, one phase 3 CABP study, and two phase 3 ABSSSI studies. The final population PK model was a three-compartment model with first-order absorption using transit compartments to account for absorption delay following oral dosing and first-order elimination. Epithelial lining fluid (ELF) concentrations were modeled as a subcompartment of the first peripheral compartment. A food effect on oral bioavailability was included in the model. Sex was the only significant covariate identified, with 15.6% lower clearance for females than males. Goodness-of-fit diagnostics indicated a precise and unbiased fit to the data. The final model, which was robust in its ability to predict plasma and ELF exposures following omadacycline administration, was also able to predict the central tendency and variability in concentration-time profiles using an external phase 3 ABSSSI data set. A population PK model, which described omadacycline PK in healthy subjects and infected patients, was developed and subsequently used to support pharmacokinetic-pharmacodynamic (PK-PD) and PK-PD target attainment assessments.

## Abstract (precis)

Omadacycline, a novel aminomethylcycline antibiotic with activity against Gram-positive and -negative organisms, including tetracycline-resistant pathogens, received FDA approval in October 2018 for the treatment of patients with acute bacterial skin and skin structure infections (ABSSSI) and community-acquired bacterial pneumonia (CABP). A previously developed population pharmacokinetic (PK) model based on phase 1 intravenous and oral PK data was refined using data from infected patients. Data from 10 phase 1 studies used to develop the previous model were pooled with data from three additional phase 1 studies, a phase 1b uncomplicated urinary tract infection study, one phase 3 CABP study, and two phase 3 ABSSSI studies.

## TEXT

Omadacycline is a novel, first-in-class aminomethylcycline that is synthesized via chemical modification of minocycline (1, 2). Omadacycline is active against Gram-positive, Gram-negative, anaerobic, and atypical pathogens (2–6) and is able to overcome efflux pump and ribosomal protection mechanisms of tetracycline resistance (2, 3, 5). In October 2018, omadacycline was approved by the United States Food and Drug Administration (U.S. FDA) for the treatment of adult patients with acute bacterial skin and skin structure infections (ABSSSI) and community-acquired bacterial pneumonia (CABP) (7).

To support the early development of omadacycline, a population pharmacokinetic (PK) model describing the disposition of omadacycline after intravenous (i.v.) or oral (p.o.) administration was constructed using data from phase 1 studies in healthy volunteers (8). The PK of omadacycline was best described by a three-compartment model with first-order absorption using transit compartments to account for a delay in p.o. absorption following administration of the tablet or capsule formulations and zero-order elimination. Results of this assessment demonstrated the impressive effect of food on the bioavailability of omadacycline, which was less than 3% when administered just before a meal. The bioavailability increased to 27% to 30% when meals were delayed 2 to 4 h postdose (8).

During late-stage development, the availability of PK data from additional studies, including those conducted in infected patients, provided the opportunity to refine the above-described population PK model. The objectives of these analyses were to refine the previously developed population PK model (8) using data from three additional phase 1 studies (9–11), one phase 1b uncomplicated urinary tract infection (uUTI) study (12), one phase 3 CABP study (13), and two phase 3 ABSSSI studies (14, 15). The second and third objectives were to characterize relationships between patient-specific covariates and omadacycline PK parameters and to apply the model to data from a third phase 3 study (16) as a means of external validation.

## RESULTS

### Data.

The final analysis data set consisted of 11,331 plasma PK samples collected from a total of 613 subjects. The majority of plasma PK samples (88.4%) were collected from subjects enrolled in phase 1 studies. Of the 613 subjects, 31 (5.1%) were enrolled in the phase 1b uUTI study, 180 (29.4%) were enrolled in phase 3 studies, and the remaining subjects were enrolled in phase 1 studies. Of the 180 patients enrolled in the phase 3 studies, 50 were enrolled in the CABP study. The remaining patients were enrolled in one of the two phase 3 ABSSSI studies. Compared to the data set used to develop the original population PK model (8), 3,772 (33.3%) of the plasma concentrations and 294 (48.0%) subjects were new. A total of 41 epithelial lining fluid (ELF) samples were available from 41 subjects, the concentrations for all of which were above the lower limit of quantitation (LLOQ) and, thus, included in the analysis. Summary statistics of baseline subject descriptors for the overall PK analysis population are presented in Table 1. The analysis population was predominantly male (71.0%) and Caucasian (70.6%), with normal renal function (mean creatinine clearance normalized to body surface area [CL_CR_] of 99.8 ml/min 1.73 m^2^) and a mean age and weight of 39.3 years and 78.4 kg, respectively.

**TABLE 1:** Summary statistics of subject demographics and clinical laboratory measures for the overall PK analysis population

Variable^*a*^	*N* (%)	Mean (SD^*c*^)	Median	Minimum	Maximum
Age (yr)	613 (100)	39.3 (14.8)	37.0	18.0	88.0
Wt (kg)	613 (100)	78.4 (14.6)	77.5	36.0	148
Ht (cm)	613 (100)	173 (9.2)	174	137	201
BSA (m^2^)	613 (100)	1.92 (0.19)	1.92	1.25	2.73
BMI (kg/m^2^)	613 (100)	26.2 (4.5)	25.6	16.0	49.3
CL_CR_ (ml/min/1.73 m^2^)	613 (100)	99.8 (28.1)	113	5.5	185
Albumin (mg/dl)	613 (100)	4.33 (0.46)	4.40	2.20	5.30
Race					
Caucasian	474 (77.3)				
Black	109 (17.8)				
Asian	15 (2.4)				
Other^*b*^	15 (2.4)				
Sex					
Male	435 (71.0)				
Female	178 (29.0)				
Presence of cirrhosis					
No	595 (97.1)				
Yes	18 (2.9)				
Presence of infection					
No	402 (65.5)				
Yes	211 (34.4)				
Presence of skin infection					
No	483 (78.8)				
Yes	130 (21.2)				
Presence of CABP infection					
No	563 (91.8)				
Yes	50 (8.2)				
Presence of uUTI					
No	582 (94.9)				
Yes	31 (5.1)				

a BSA, body surface area; BMI, body mass index.

b Includes American Indian, Alaska native, native Hawaiian, or other Pacific islander.

c SD, standard deviation.

The external validation data set consisted of 202 patients enrolled in a third phase 3 ABSSSI study (16). Summary statistics of baseline subject descriptors for the external validation data set population are provided in Table S1 in the supplemental material. Similar to the analysis data set, the external validation data set population was predominantly male (68.8%) and Caucasian (86.6%), with normal renal function (mean CL_CR_ of 106 ml/min/1.73 m^2^) and a mean age and weight of 41.5 years and 81.5 kg, respectively.

### Population pharmacokinetic model.

During the process to develop the structural model, it was confirmed that the previously developed three-compartment model, with first-order absorption using transit compartments to account for a delay in p.o. absorption following administration of the tablet or capsule formulations and first-order elimination (8), best described the time course of omadacycline in plasma for the final analysis data set.

ELF concentrations were modeled as a subcompartment of the first peripheral compartment. In the model, omadacycline distributes between the central compartment and the first peripheral compartment in a manner similar to that of the central compartment and ELF compartment; however, ELF concentrations were scaled using a “FRAC” term (Table 2). The FRAC term allowed for the ELF to be estimated as a fraction of the concentration in the first peripheral compartment rather than as a fraction of the amount in the first peripheral compartment. FRAC was incorporated to reduce the bias observed in the ELF goodness-of-fit plots.

**TABLE 2:** Final parameter estimates for the final population PK model

Parameter^*a*^	Final estimate	%SEM
CL (liters/h)	10.3	0.682
Proportional change in females	−0.156	12.0
*V_c_* (liters)	21.1	2.20
CL_d1_ (liters/h)	101	2.20
Proportional change in females	0.500	27.6
*V_p_*_1_ (liters)	79.9	0.0842
Proportional change in females	−0.176	16.9
CL_d2_ (liters/h)	21.3	0.242
*V_p_*_2_ (liters)	129	1.45
Proportional change in females	−0.271	9.45
ka (h^−1^)	1.74	1.55
*F*_0_	0.00663	4.99
*F*_max_	0.252	0.996
Proportional decrease for Capsugel capsules or freebase capsules, >200 mg	−0.280	21.8
AMTIME_50_ (h)	0.568	0.0567
Proportional increase for consuming food predose	1.68	8.15
Proportional increase for consuming food with dairy products predose	3.59	4.48
γ	1.73	0.484
ELF FRAC^*b*^	1.63	5.69
ω^2^ for CL	0.0497 (22.3% CV)	7.72
ω^2^ for *V_c_*	0.885 (94.1% CV)	10.9
ω^2^ for CL_d1_	0.423 (65.0% CV)	10.8
ω^2^ for *V_p_*_1_	0.0776 (27.9% CV)	10.6
ω^2^ for *V_p_*_2_	0.0759 (27.5% CV)	9.59
ω^2^ for F	0.154 (39.2% CV)	5.28
ω^2^ for ka	0.0599^*c*^ (24.5% CV)	4.79
IOV for ka	0.0599^*c*^ (24.5% CV)	4.79
IOV for F	0.0495 (22.2% CV)	3.21
Covariance (CL, CL_d1_)	−0.0415 (*r*^2^ = 0.0819)	23.3
Covariance (CL, *V_p_*_2_)	0.0258 (*r*^2^ = 0.176)	16.4
σ^2^_CCV, plasma_	0.0217 (14.7% CV)	0.0399
σ^2^_additive, plasma_	0.00145 (0.0381 SD)	0.163
σ^2^_CCV, ELF_	0.206 (45.4% CV)	24.5
σ^2^_additive, ELF_	0.000403 (0.0201 SD)	Fixed

a AMTIME_50_, the absolute time of food consumption relative to dosing at which 50% of *F*_max_ could be achieved; *F*, bioavailability; *F*_0_, absolute bioavailability; ka, absorption rate constant; IOV, interoccasion variability; γ, Hill function sigmoidicity or shape factor; ω^2^, variance on an interindividual variability term; σ^2^, variance on a residual variability term.

b FRAC represents a proportionality term allowing for scaling of the concentration of omadacycline in *V_p_*_1_ to a concentration in ELF.

c A single parameter was used to describe both ka IIV and IOV.

The final model was confirmed to be a linear, three-compartment model with zero-order i.v. input and first-order absorption using transit compartments to account for a delay in oral absorption following the administration of the tablet or capsule formulations. In the final model, oral bioavailability decreased when food was administered before or after oral dosing. This effect was greater when food was taken before oral administration of omadacycline or when the food contained dairy products. The final parameter estimates and their associated precision (percent standard errors of the means [%SEM]) for the final population PK model describing the time course of omadacycline plasma and ELF PK profiles are provided in Table 2. The mean (percent coefficient of variation [%CV]) of the clearance (CL) was 10.3 (22.3%) and 8.69 liters/h (22.3%) for males and females, respectively. The interindividual variability (IIV) was relatively low for all parameters for which IIV was estimated, with the exception of the central volume of distribution (*V_c_*) and the distributional clearance of the first peripheral compartment (CL_d1_; %CV of 94.1% and 65.0%, respectively). The precision of the parameters was high, with %SEM values generally under 20% and none higher than 27.6%.

The results of the covariate analysis demonstrated that sex was the only significant covariate identified. Females had a 15.6% lower clearance, a 50% higher distributional clearance of CL_d1_, a 17.6% lower volume of distribution for the first peripheral compartment (*V_p_*_1_), and a 27.1% lower volume of distribution for the second peripheral compartment (*V_p_*_2_) than males. Simulations of typical male and female subjects using the final population PK model showed that the maximum concentration (*C*_max_) was 9% lower, the minimum concentration (*C*_min_) was 25% higher, and the area under the concentration-time curve (AUC) from time zero to infinity (AUC_0–inf_) was 15.6% higher for females than males following i.v. administration of a single 100-mg omadacycline dose. Similar findings were evident for oral administration.

**FIG 1:**
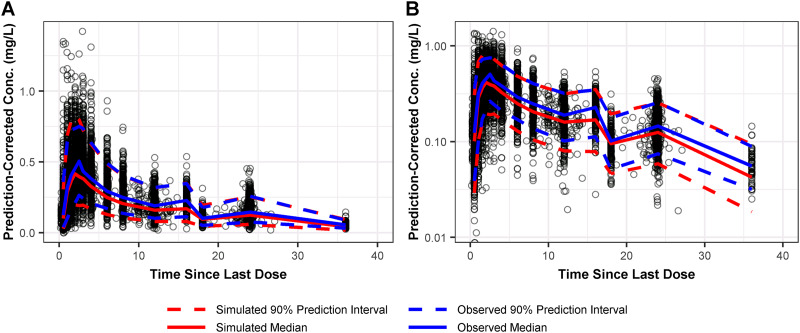
Prediction-corrected visual predictive check of observed and simulated omadacycline plasma concentrations on linear (A) and log (B) scales for the final population PK model.

Plasma goodness-of-fit diagnostics for the final population PK model indicated a precise and unbiased fit to the data, as seen in Fig. S1. There was excellent agreement between the observed plasma omadacycline concentrations and both the population-predicted (coefficient of determination [*r*^2^] = 0.743) and individual-predicted (*r*^2^ = 0.961) concentrations. Prediction-corrected visual predictive check (PC-VPC) plots showed the majority of the observed plasma PK data were contained within the prediction interval, as seen in Fig. 1. Additionally, the observed median and 90% prediction interval were similar at most time points to the simulated median and 90% prediction interval, suggesting that the final population PK model provided a precise and unbiased fit of the plasma PK data. Given this, the final population PK model was expected to provide robust and reliable estimates of omadacycline plasma exposure.

**FIG 2:**
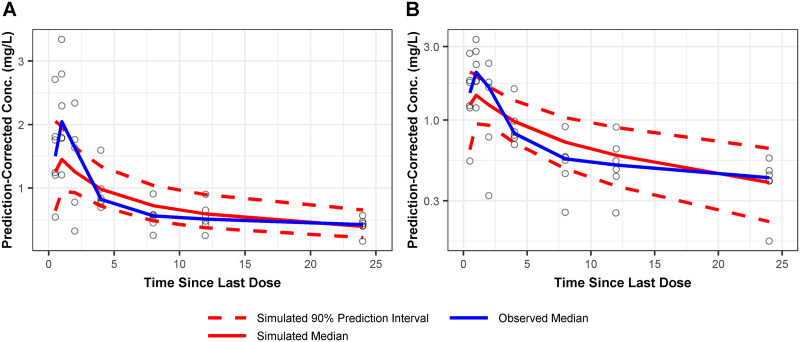
Prediction-corrected visual predictive check of observed and simulated omadacycline ELF concentrations on linear (A) and log (B) scales for the final population PK model. The 90% prediction interval for the observed data is not displayed, given that there were no more than six ELF PK samples per time point.

All ELF parameters were modeled as fixed effects, as there was only one ELF sample per patient. As a result, IIV could not be determined. Goodness-of-fit and PC-VPC plots for the final population PK model for the ELF PK data are shown in Fig. S2 and Fig. 2, respectively. Although early time points were slightly underpredicted, observations were equally dispersed above and below the model-predicted ELF concentration-time curve (bottom left of Fig. 2 and Fig. S2). Given this and the lack of an IIV term on the ELF parameter, the model was deemed to adequately describe the limited ELF data. Using an omadacycline protein binding value of 21% (17) and the final population PK model, the total-drug ELF:free-drug plasma penetration ratio was calculated to be 2.06.

**FIG 3:**
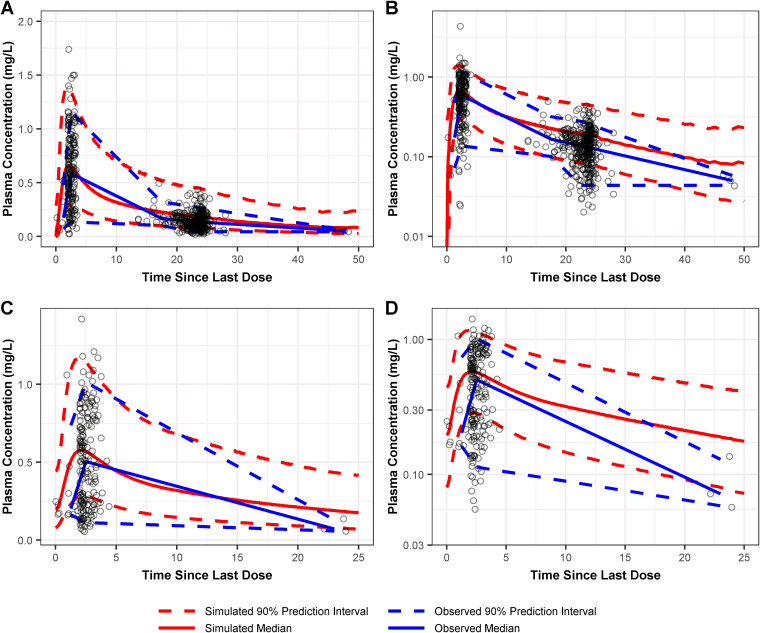
Prediction-corrected visual predictive check of observed and simulated omadacycline plasma concentrations on linear and log scales following administration of 450 mg omadacycline p.o. q24h (A and B, respectively) and 300 mg p.o. q24h (C and D, respectively) from the external validation data set for the final population PK model.

External validation based on data from a separate phase 3 ABSSSI study (16) indicated that the central tendency and distribution of concentration-time profiles were well predicted by the final model. The resultant PC-VPC plots are provided in Fig. 3 and show similar profiles of the median simulated concentration and the median observed concentration. Additionally, the 90% prediction interval for the simulated concentrations was similar to that of the observed concentrations from the external validation data set, indicating that the model could predict the magnitude of PK variability across patients with ABSSSI.

## DISCUSSION

The objectives of the analyses were to refine a previously developed population PK model (8) for omadacycline, to assess the impact of patient-specific covariates on omadacycline exposures, and to externally validate the final model, utilizing data obtained from an additional phase 3 study.

The population PK model describing omadacycline PK in healthy subjects and infected patients was found to be a three-compartment model with first-order absorption using transit compartments and first-order elimination. ELF concentrations were modeled as a subcompartment of the first peripheral compartment. The final parameter estimates were largely comparable to the estimates of the original model (8). However, both the maximum increase in absolute bioavailability in the absence of food (*F*_max_) and *V_p_*_1_ had marked increases in IIV. This was not surprising, given the increased PK variability that has been observed for infected patients compared to that of healthy volunteers (18–21).

In contrast to the structure of the model, the covariance terms in the final model were not the same as those in the original model (8). While the previous model contained covariance terms for CL-*V_c_*, *V_c_-V_p_*_2_, and CL-*V_p_*_2_, the updated model only retained CL-*V_p_*_2_ and added a new term for CL-CL_d1_. Excluding relationships between each of *V_c_* and the presence of cirrhosis and CL_CR_ (which were in the previous model), and the relationships between sex and all systemic PK parameters included in the refined model, the remaining covariate relationships were the same between the models. The presence of cirrhosis was evaluated in all steps of the forward selection process due to the results of univariable screening analyses, which suggested a relationship between omadacycline *V_c_* and the presence of cirrhosis. However, the relationship was never found to be statistically significant when evaluated (*P* > 0.01). The previous population PK data set had a CL_CR_ minimum value of 52.8 ml/min/1.73 m^2^, while the updated data set had a much wider CL_CR_ range and a lower minimum value of 5.5 ml/min/1.73 m^2^. Thus, the relationship between CL and CL_CR_ was able to be more robustly assessed and, as a result, was not found to be significant.

The development of the above-described population PK model required accurate characterization of the impact of the time of food consumption relative to dosing on bioavailability. In the final population PK model, bioavailability was parameterized as a function of the absolute time of food consumption relative to dosing using a Hill-type function. The estimated intercept or predicted bioavailability when a dose was given at the same time as food was 0.00663. The *F*_max_ was 0.252, which began to plateau when food was consumed at least 2 h postdose (*F* = 0.233) and was near maximum by 4 h postdose. Since bioavailability was more sensitive to food consumption predose than when food was consumed at the same time postdose, the absolute time of food consumption relative to dosing at which 50% of *F*_max_ could be achieved (AMTIME_50_), which was equal to 0.568 h, was adjusted accordingly. When food was consumed before dose administration, AMTIME_50_ was increased to 1.52 h. Bioavailability decreased further when the food contained dairy products. For this situation, the AMTIME_50_ was increased to 2.61 h. Therefore, consuming food between approximately 1.5 h before dosing and 0.5 h after dosing reduces omadacycline bioavailability by approximately 50%, from a value of 0.26 to a value of 0.14. These findings support the recommendations in the U.S. FDA package insert for omadacycline p.o. administration, which advise fasting for at least 4 h before taking omadacycline and waiting for at least 2 h after administration before consuming any food or fluids (except water) and 4 h after administration before consuming dairy products, antacids, or multivitamins (7).

The assessment of the impact of patient-specific covariates on omadacycline exposures, which represented the second objective of these analyses, demonstrated that the overall effect of sex was not impressive. Using the final model, simulations of typical male and female subjects showed that AUC_0–inf_ increased by 15.6%, *C*_max_ decreased by 9%, *C*_min_ increased by 25%, and CL decreased by 15.6% for females relative to males following i.v. administration of a single 100-mg omadacycline dose. The addition of these sex-based relationships reduced the Bayesian shrinkage for all parameters, indicating that sex aids in the estimation of the IIV in omadacycline PK parameters. However, a significant decrease in IIV was not observed for any parameters after the addition of sex as a covariate; thus, the net effect on the omadacycline PK profile was minimal.

An external validation of the final population PK model, which represented the third objective of these analyses, was conducted utilizing data obtained from an additional phase 3 study conducted in patients with ABSSSI. Using the final model presented herein, the distribution of omadacycline concentration-time profiles among patients in the phase 3 study not included in model development was predicted reasonably well. Validation of a population PK model using external data is a useful approach to ensure that the model has good predictive performance (22, 23). The results of the external validation described herein indicated that the predictive performance of the model was robust. However, a limitation of these analyses was that the study data used for external validation only provided plasma concentrations after p.o. rather than i.v. administration. Thus, additional study data are needed to further validate the final population PK model across multiple routes of administration.

The development of a population PK model that described the disposition of omadacycline in both plasma and ELF allowed for the opportunity to characterize the penetration of drug to the effect site. The total-drug ELF:free-drug plasma penetration ratio for omadacycline based on day 4 AUC from time zero to 24 h (AUC_0–24_) was 2.06 in the current analysis, which is similar to the previously reported ratio of 1.84 determined via noncompartmental analysis (9). The evaluation of such data was associated with certain limitations. As only one bronchoalveolar lavage (BAL) fluid sample was obtained for each subject, IIV could not be calculated. Additionally, ELF samples were only collected from healthy subjects. However, despite the first limitation, the estimation of omadacycline ELF exposure using a population PK model, which was developed by comodeling plasma and ELF concentration data, represented a robust approach. With regard to the second limitation, while data evaluating ELF exposures for antimicrobial agents in infected patients are limited (24–28) and more challenging to obtain, future studies in omadacycline-treated patients would be useful to determine if ELF exposure in patients is different from that in healthy volunteers.

In summary, the disposition of omadacycline was found to be best described by a three-compartment model with first-order absorption using transit compartments to account for a delay in oral absorption and first-order elimination. Model-predicted exposures in omadacycline-treated patients were accurate and precise, important criterion for PK-PD analyses for efficacy and safety. Monte Carlo simulations utilizing the final population PK model would be expected to generate reliable omadacycline exposures in the target patient population. Accordingly, the application of the population PK model described herein for subsequent PK-PD and PK-PD target attainment analyses provided support for omadacycline dose selection and the evaluation of interpretive criteria for *in vitro* susceptibility testing for omadacycline against relevant pathogens (29, 30).

## MATERIALS AND METHODS

### Data.

Ten phase 1 studies were used to construct the previous population PK model (8). Three phase 1 studies and a phase 1b study were added to this data set for the current analyses. A summary of dosing regimens, including the route of administration and formulation type for capsule and tablets, sampling strategies, and the number of subjects or patients considered for the population PK analyses by study is provided in Table S2 in the supplemental material. In the first phase 1 study, omadacycline at 300, 450, or 600 mg p.o. was administered to healthy subjects once daily for 5 days under fasted conditions (10). Plasma PK sampling in this study was performed intensively on days 1 and 5. In the second study, healthy adults and those with end-stage renal disease (ESRD) were administered a single omadacycline 100-mg i.v. dose (11). Intensive plasma PK sampling was performed during the first 24 h, with additional samples drawn at 48 and 68 h. In the third study, healthy adults were administered omadacycline at 100 mg i.v. at 0, 12, 24, 48, and 72 h (9). Plasma PK sampling was performed intensively on day 4. Additionally, BAL fluid samples were obtained once per subject at various time points on day 4 up to 24 h after the last dose was administered.

In the phase 1b uUTI study, patients in group 1 received 200 mg omadacycline i.v. on day 1, followed by 300 mg omadacycline p.o. every 24 h (q24h) on days 2 through 5 (12). Patients in group 2 received 300 mg omadacycline p.o. every 12 h (q12h) on day 1, followed by 300 mg p.o. q24h on days 2 through 5. Patients in group 3 received 450 mg omadacycline p.o. q12h on day 1, followed by 450 mg p.o. q24h on days 2 through 5. Intensive plasma PK sampling was conducted on days 1 and 5. All oral doses were administered under fasted conditions.

In the phase 3 CABP study, patients were administered 100 mg omadacycline i.v. q12h on day 1, followed by 100 mg i.v. with the option to switch to 300 mg p.o. q24h after at least 3 days, for a total treatment duration of 7 to 14 days (13). Up to four PK samples per patient were collected between days 1 and 7 using two different sampling schedules. For both schedules, the first sample was drawn 3 to 5 h after the start of the first infusion, the third sample was drawn within 30 min before the eighth infusion or oral dose, and the fourth sample was drawn within 1 to 3 h after the eighth infusion or oral dose. For the first schedule, the second sample was drawn within 30 min before the start of the second infusion, while for the second schedule, it was drawn within 30 min before the start of the fourth or fifth infusion or oral dose.

In the two phase 3 ABSSSI studies included in the current analyses and the additional phase 3 ABSSSI study data used for model validation (also summarized in Table S2), three different omadacycline dosing regimens were administered. In the first study, patients received 100 mg omadacycline i.v. q24h for 4 to 7 days, followed by 300 mg p.o. q24h for up to a total of 14 days (14). All subjects received an i.v. infusion every 12 h to maintain the study blind. Plasma samples for PK analysis were collected predose (within 24 h before the first infusion) and at 1, 3, 6, 12, and 24 h after the start of the first infusion. Additional plasma samples were drawn predose for the seventh infusion (72 h after the start of the first infusion) and 1 h after the start of the seventh infusion. Samples were also drawn at the end of i.v. treatment, periodically during p.o. therapy, and at the end of treatment. In the second study, patients received 100 mg omadacycline i.v. q12h for two doses, followed by 100 mg i.v. q24h, with a possible switch to 300 mg p.o. q24h for a total of up to 14 days (15). Both once-daily i.v. and p.o. omadacycline regimens of the study utilized a placebo as the second daily dose to maintain the study blind. PK sampling was performed using two different sampling schemes. Sample schedule A had samples collected within 3 to 5 h after the start of the first i.v. dose, immediately (within 30 min) before the start of the second i.v. infusion, immediately before the 13th dose, whether i.v. or p.o., and within 1 to 3 h after the 13th dose. Sampling schedule B had samples collected within 3 to 5 h after the start of the first i.v. infusion immediately before the start of the fifth or seventh infusion, immediately before the start of the 13th dose, whether i.v. or p.o., and within 1 to 3 h after the 13th dose. In the third study, patients received 450 mg omadacycline p.o. q24h for two doses, followed by 300 mg p.o. q24h, for a total treatment duration of 7 to 14 days (16). The double-blind nature of the study required a placebo to be administered 12 h after the omadacycline dose, as the comparator regimen was administered q12h. Four PK samples were collected between days 2 and 3; samples were drawn immediately before the first dose on day 2, within 2 to 4 h after the first dose on day 2, immediately before the first dose on day 3, and within 2 to 4 h after the first dose on day 3. PK data from this third study were only used for external model qualification, as these data were not available during model development.

Omadacycline plasma concentrations were determined using a validated liquid chromatography-tandem mass spectrometry (LC-MS/MS) assay with an LLOQ of 20 ng/ml. Omadacycline concentrations in BAL fluid were determined using a validated LC-MS/MS assay with an LLOQ of 0.05 ng/ml. The omadacycline ELF concentration was determined by dividing the omadacycline BAL fluid concentration by the urea BAL fluid concentration and then multiplying by the urea plasma concentration obtained at the same time point (9, 31).

### Population pharmacokinetic model.

The previously developed population PK model was a three-compartment model with first-order absorption using transit compartments to account for a delay in oral absorption and first-order elimination (8). This model served as a starting point for model development and refinement. Candidate population PK models were fit to the pooled PK data using NONMEM version 7.2, implementing the first-order conditional estimation method with interaction (FOCE-I). The following criteria were used to evaluate candidate population PK models: examination of individual and population mean parameter estimates and their precision, graphical examination of goodness-of-fit plots, reduction in residual variability and interindividual variability, and comparison of the minimum value of the objective function for nested models or Akaike’s information criterion for nonnested models if necessary.

Interindividual variability for each PK parameter was described, where possible, using an exponential error model assuming a log-normal distribution. A combined additive plus constant coefficient of variation (CCV) error model was used to describe plasma residual variability, and a CCV error model was used to describe the ELF residual variability.

The ability of subject demographics (race, age, and sex), various body size measures, albumin level, renal function, presence of cirrhosis, and the presence of various infections to explain IIV on selected omadacycline PK parameters then was explored using stepwise forward selection (α = 0.01) and backward elimination (α = 0.001) procedures.

In addition to traditional goodness-of fit plots, a PC-VPC was used to evaluate the ability of the final model to adequately describe the observed omadacycline plasma and ELF PK data used for model development. The final model was also qualified using an external validation approach in a similar manner using PC-VPC plots to compare model-based predictions to observed data from the third phase 3 ABSSSI study, which was not utilized for model development.

The individual *post hoc* PK parameter estimates obtained from the final model were utilized to simulate predicted plasma omadacycline concentration-time data in the first 48 h of therapy. The *C*_max_ was the highest predicted concentration. The *C*_min_ was the lowest predicted concentration. AUC was calculated via numerical integration of the concentration-time profile. A Monte Carlo simulation of subjects receiving 100 mg of omadacycline by i.v. infusion at 0, 12, 24, 48, and 72 h then was performed using the final population PK model to determine ELF penetration. Cumulative total-drug AUC for both plasma and ELF was calculated by numerical integration for each subject. Total-drug plasma and ELF AUC_0–24_ values on day 4 were computed for each subject using the cumulative AUC. Free-drug plasma day 4 AUC_0–24_ was calculated assuming an omadacycline protein binding estimate of 21.0% (17). Day 4 total-drug ELF:free-drug plasma penetration ratios then were calculated by dividing the total-drug ELF day 4 AUC_0–24_ by the free-drug plasma day 4 AUC_0–24_.

## Supplementary Material

Supplemental file 1
