# Supplementary material for: Population Pharmacokinetic Analyses for Omadacycline Using Phase 1 and 3 Data
Source: Antimicrob Agents Chemother. 2020 Jun 23;64(7):e02263-19. doi: 10.1128/AAC.02263-19 (PMC7318031; doi:10.1128/AAC.02263-19)
Supplement: Supplemental file 1 [file AAC.02263-19-s0001.pdf]

Table S1. Summary statistics of patient demographics, clinical laboratory measures, and disease-related indices for the external validation dataset

Variable		N (%)	Mean (SD)	Median	Minimum	Maximum
Age (yr)		202 (100)	41.5 (11.9)	39.5	20	78
Weight (kg)		202 (100)	81.5 (18.0)	79.8	41.7	167
Height (cm)		202 (100)	172 (9.14)	172	137	194
BSA (m^2)		202 (100)	1.94 (0.20)	1.94	1.39	2.6
BMI (kg/m^2)		202 (100)	27.6 (6.62)	26.5	16.3	71.3
CLcr (mL/min/1.73 m^2)		202 (100)	106 (33.0)	103	39	214
Albumin (mg/dL)		202 (100)	4.01 (0.350)	4.00	2.6	5.00
Race	Caucasian	175 (86.6)	—	—	—	—
	Black	16 (7.92)	—	—	—	—
	Asian	2 (0.99)	—	—	—	—
	Other[a]	9 (4.46)	—	—	—	—
Sex	Male	139 (68.8)	—	—	—	—
	Female	63 (31.2)	—	—	—	—
Presence of cirrhosis	No	212 (100)	—	—	—	—
	Yes	0 (0)	—	—	—	—

Note: SD = Standard deviation.
a. Includes American Indian or Alaska Native and Native Hawaiian or other Pacific Islander.

Table S2. Summary of omadacycline clinical studies used in the population PK analyses

Study [Reference]	Phase	Title	Subjects/Patients (n)[a]	Omadacycline dosing regimens	Scheduled plasma PK sample collection times
PTK 0796-OBAV-0502	1	A placebo-controlled, randomized, double-blind, Phase 1 study in healthy male subjects to investigate the safety, tolerability and bioavailability of ascending single oral doses of PTK 0796	63	Single oral 50, 100, 150, 200, 300, 400, or 600 mg omadacycline freebase capsule in parallel groups	Pre-dose, 0.5, 1, 1.5, 2, 3, 4, 6, 8, 12, 18, 24, 36, and 48 hours post-dose
PTK 0796-BEQU-0801	1	A placebo–controlled, randomized, double-blind, Phase 1 study to investigate the bioequivalence and safety of oral formulations of PTK 0796 freebase capsules and PTK 0796 tosylate salt tablets and to determine the bioavailability of PTK 0796 tosylate salt tablets in healthy subjects	32	Group A: Single oral 200 mg omadacycline freebase capsule<br><br>Group B: Single oral omadacycline tosylate tablet, then a single 100 mg IV infusion of omadacycline HCl over 0.5 hours after a 7-day minimum washout	Pre-dose, 0.5, 1, 1.5, 2, 3, 4, 6, 8, 12, 18, 24, 48, 72, and 96 hours post-dose in each period
PTK 0796-BEQV-0806	1	A randomized, open label, parallel group, Phase 1 study to compare the pharmacokinetics of two oral capsule[b] formulations of PTK 0796 (freebase and tosylate salt) in healthy adults	30	Single oral 200 mg omadacycline freebase or tosylate salt capsules (Capsugel or Qualcaps) in fed/fasted state in 5 parallel groups	Pre-dose, 0.25, 0.5, 1, 1.5, 2, 3, 4, 6, 8, 12, 18, and 24 hours post-dose in each period
PTK 0796-BAVA-0810	1	A Phase 1 study to evaluate the pharmacokinetics and safety of three dose levels of oral PTK 0796 tosylate salt and determine the bioequivalence of the proposed therapeutic dose in healthy young subjects	40	Part A: Single oral 250 or 300 mg omadacycline tosylate tablets in parallel in fed/fasted state<br><br>Part B: Single oral 300 mg omadacycline tosylate tablet and 100 mg IV infusion of omadacycline tosylate over 0.5 hours separated by minimum 6-day washout	Part A: Pre-dose, 0.5, 1, 1.5, 2, 3, 4, 6, 8, 12, 18, and 24 hours post-dose<br><br>Part B: Pre-dose, 0.5, 1, 1.5, 2, 3, 4, 6, 8, 12, 18, and 24, 48, and 72 hours post-dose
PTK 0796-MDOR-0901	1	A Phase 1 study to evaluate the pharmacokinetics and safety of single and multiple oral doses of PTK 0796 tablets	24	Oral 300 mg omadacycline tosylate tablet QD for 5 days	Day 1: Pre-dose, 0.5, 1, 1.5, 2, 3, 4, 6, 8, 12, 18, and 24 hours post-dose<br><br>Days 2-4: Pre-dose<br><br>Day 5: 0.5, 1, 1.5, 2, 3, 4, 6, 8, 12, 18, and 24, 48, and 72 hours post-dose

Study [Reference]	Phase	Title	Subjects/Patients (n)[a]	Omadacycline dosing regimens	Scheduled plasma PK sample collection times
CPTK796A2103	1	A randomized, open-label, five period, complete cross over study to evaluate the effects of food content and timing on the relative bioavailability of a single oral dose of PTK796 in healthy subjects	40	Single oral 300 mg omadacycline tosylate tablet in fed/fasted states (5-period crossover)	Pre-dose, 0.5, 1, 1.5, 2, 2.5, 3, 4, 6, 8, 12, 24, and 48 hours post-dose in each period
CPTK796A2201	1	An open-label, fixed sequence study to evaluate the pharmacokinetics and safety of single IV and oral doses of PTK796 in subjects with mild, moderate, and severe hepatic impairment compared to healthy subjects with normal liver function	30	Mild hepatic impairment and matching controls: Single dose of 100 mg IV omadacycline tosylate infused over 0.5 hours, followed by single dose of 300 mg PO omadacycline tosylate tablet after minimum 7-day washout<br><br>Moderate hepatic impairment and matching controls: Single IV infusion of 50 mg omadacycline tosylate over 0.5 hours, followed by single dose of 150 mg PO omadacycline tosylate tablet after minimum 7-day washout<br><br>Severe hepatic impairment: Single IV infusion of 50 mg omadacycline tosylate over 0.5 hours	Pre-dose, 0.25[c], 0.5, 1, 1.5, 2, 3[d], 4, 6, 8, 12, 24, 48, 72, and 96 hours post-dose in each period
CPTK796A2104	1	A randomized, open-label, four period, crossover study to evaluate the single dose pharmacokinetics, safety, and tolerability of multiple formulations of PTK796 in healthy subjects	24	Single IV infusion of 100 mg omadacycline tosylate over 0.5 hours, single oral 300 mg omadacycline tosylate tablet, single 300 mg slow dissolution tablet, and 300 mg oral solution[e], each separated by a minimum 7-day washout	Pre-dose, 0.25[c], 0.5, 1, 1.5, 2, 2.5[d], 3, 4, 6, 8, 12, 24, and 48 hours post-dose in each period
CPTK796A2101	1	An open-label study to assess the absorption, distribution, metabolism and elimination of [^{14}C]-labeled PTK796 in healthy male subjects following a single oral dose of 300 mg PTK796	6	Single oral 300 mg radiolabeled [^{14}C] omadacycline	Pre-dose, 0.5, 1, 1.5, 2, 2.5, 3, 4, 8, 12, 24, 36, 48, 72, 96, 120, and 144 hours post-dose

Study [Reference]	Phase	Title	Subjects/Patients (n)[a]	Omadacycline dosing regimens	Scheduled plasma PK sample collection times
PTK0796-FDEF-15101	1	A Phase 1, randomized, open-label, four period, complete crossover study to evaluate the relative bioavailability of a single oral dose of omadacycline in healthy subjects following the consumption of food	32	Single oral 300 mg omadacycline tosylate tablets in each period under different fasting conditions	Pre-dose, 0.5, 1, 1.5, 2, 2.5, 3, 4, 6, 8, 12, 16, and 24 hours post-dose in each period
PTK0796-RENL-15102 [1]	1	An open-label study to evaluate the pharmacokinetics and safety of a single IV dose of omadacycline in renally impaired adult subjects as compared to matched healthy adult subjects	16	Single IV infusion of 100 omadacycline tosylate over 0.5 hours[f]	Pre-dose, 0.5, 1, 2, 3, 4, 5[g], 6, 10, 24, 48, and 68 hours post-dose
PTK0796-BAL-15104 [2]	1	An open-label, parallel group, multiple IV dose study to assess intra-pulmonary steady-state concentrations of omadacycline and tigecycline in healthy adult subjects	41	Multiple-dose IV infusion of 100 omadacycline tosylate over 0.5 hours (doses administered at 0, 12, 24, 48, and 72 hours)	Day 4: Pre-dose, 0.5, 1, 1.5, 2, 3, 4, 6, 8, 12, and 24 hours post-dose<br><br>Note: BAL sample also collected at various time points on Day 4
PTK0796-MDPO-16105 [3]	1	A Phase 1, randomized, double-blind, three period, crossover study to evaluate safety, tolerability, and pharmacokinetics of multiple oral doses of omadacycline or placebo in healthy adult subjects	26	300, 450, or 600 mg omadacycline tosylate tablets PO q24h for 5 days	Days 1 and 5: Pre-dose, 0.5, 1, 1.5, 2, 2.5, 3, 4, 6, 8, 12, 16, and 24 hours post-dose
PTK0796-UUTI-15103 [4]	1b	A Phase 1b study to evaluate the safety and pharmacokinetics of omadacycline in female adults with cystitis	31	Group 1: omadacycline 200 mg IV on Day 1 followed by omadacycline tosylate tablets 300 mg PO q24h Days 2-5<br><br>Group 2: omadacycline tosylate tablets 300 mg PO q12h on Day 1 followed by q24h Days 2-5<br><br>Group 3: omadacycline tosylate tablets 450 mg PO q12h on Day 1 followed by q24h Days 2-5	Day 1: Pre-dose, 0.75, 1, 2, 3, 4, 6, 8, and 12 hours post-dose<br><br>Days 2-4: Pre-dose<br><br>Day 5: Pre-dose, 0.5, 1, 2, 3, 4, 6, 8, 12, and 24 hours post-dose

Study [Reference]	Phase	Title	Subjects/Patients (n)[a]	Omadacycline dosing regimens	Scheduled plasma PK sample collection times
PTK0796-CABP-1200 [5]	3	A Phase 3, randomized, double-blind, multi-center study to compare the safety and efficacy of omadacycline IV/PO to moxifloxacin IV/PO for treating adult subjects with community-acquired bacterial pneumonia (CABP)	50	100-mg IV dose of omadacycline tosylate infused over 0.5 hours q12h on Day 1 followed by 100 mg IV q24h for up to a total of 14 days of treatment; optional switch to 300 mg PO q24h after at least 3 days of IV treatment	Schedule A: 3-5 hours after the first infusion, immediately prior to the second infusion, immediately prior to the eighth dose (IV or PO), and 1-3 hours after the eighth dose<br><br>Schedule B: 3-5 hours after the first infusion, immediately prior to the fourth or fifth dose (IV or PO), immediately prior to the eighth dose (IV or PO), and 1-3 hours after the eighth dose
PTK0796-CSSI-0804[h] [6]	3	A randomized, evaluator-blinded, Phase 3 study to compare the safety and efficacy of omadacycline with linezolid in the treatment of adults with complicated skin and skin structure infection	57	100 mg IV dose of omadacycline tosylate infused over 0.5 hours q24h for 4-7 days followed by 300 mg oral dose of omadacycline tosylate tablets q24h for up to a total of 14 days of treatment	During IV Dosing: Pre-dose, 1, 3, 6, 12 (pre-dose for infusion 2), 24 (pre-dose for infusion 3), 72 (pre-dose for infusion 7), and 73 (1 hour after start of infusion 7) hours after the start of the first infusion; additional sample collected at the end of IV treatment<br><br>During oral dosing: sporadically and at the end of treatment
PTK0796-ABSI-1108 (OASIS-1) [7]	3	A Phase 3, randomized, double-blind, multi-center study to compare the safety and efficacy of omadacycline IV/PO to linezolid IV/PO for treating adult subjects with acute bacterial skin and skin structure infection	80	100 mg IV dose of omadacycline tosylate infused over 0.5 hours q12h on Day 1 followed by 100 mg IV q24h for up to a total of 14 days of treatment; optional switch to 300 mg PO q24h after at least 3 days of IV treatment	Schedule A: within 3-5 hours after start of first IV dose, immediately prior to second IV dose, immediately prior to 13th dose (IV or PO), and within 1-3 hours after 13th dose<br><br>Schedule B: within 3-5 hours after start of first IV dose, immediately prior to fifth or seventh IV dose, immediately prior to 13th dose (IV or PO), and within 1-3 hours after 13th dose

Study [Reference]	Phase	Title	Subjects/Patients (n)[a]	Omadacycline dosing regimens	Scheduled plasma PK sample collection times
PTK0796-ABSI-16301 (OASIS-2)[i] [8]	3	A Phase 3, randomized, double-blind, multi-center study to compare the safety and efficacy of oral omadacycline to oral linezolid for treating adult subjects with acute bacterial skin and skin structure infection (ABSSSI)	202	Omadacycline tosylate tablets 450 mg PO q24h for 2 doses followed by 300 mg PO q24h for a total duration of 7-14 days	Immediately prior to the dose on Day 2, 2-4 hours after the dose on Day 2, immediately prior to the dose on Day 3, and within 2-4 hours after the dose on Day 3

Note: ABSSSI, acute bacterial skin and skin structure infection; BAL, bronchoalveolar lavage; CABP, community-acquired bacterial pneumonia; ESRD, end-stage renal disease; h, hour; IV, intravenous; mg, milligrams; PK, pharmacokinetics; PO, oral; q12h, every 12 hours; q24h, every 24 hours; uUTI, uncomplicated urinary tract infection.

a. Represents the number of subjects/patients considered for the PK population analysis, which included those who received at least one dose of omadacycline and had PK data available. This count by study includes any subjects/patients with outlier samples or samples with concentrations below the limit of quantitation and those with any other missing information that were subsequently evaluated for exclusion.

b. Subjects who received the Capsugel formulation were coded separately from subjects given Qualcaps formulation.

c. The PK sample was collected only following IV omadacycline administration.

d. The PK sample was collected only following oral omadacycline administration.

e. PK data collected from the study periods in which the slower dissolution tablet and the oral solution of omadacycline was administered were not utilized in the population PK analysis.

f. Patients with ESRD received two doses, one 60-90 minutes pre-dialysis and one 0-2 hours post dialysis, that were separated by a 10-20 day washout period.

g. The PK sample was collected only in ESRD patients who were administered omadacycline prior to dialysis.

h. Subjects who did not fast overnight or ate within 2 hours of oral omadacycline administration were excluded from the analysis.

i. Data from Study PTK0796-ABSI-16301 used for external validation and not for the development of the population PK model.

Figure S1. Plasma goodness-of-fit plots for the final population PK model

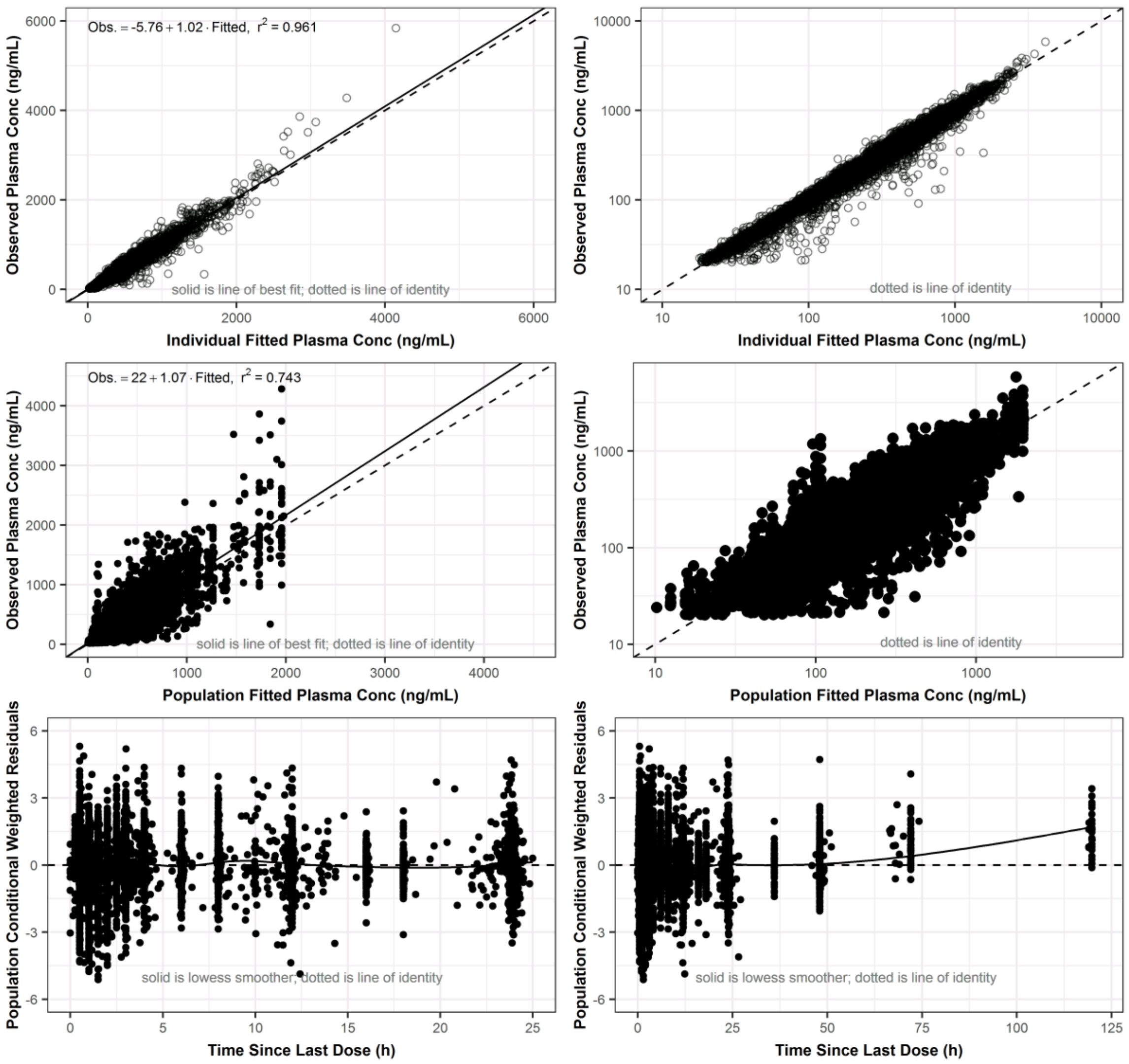

Figure S2. ELF goodness-of-fit plots for the final population PK model

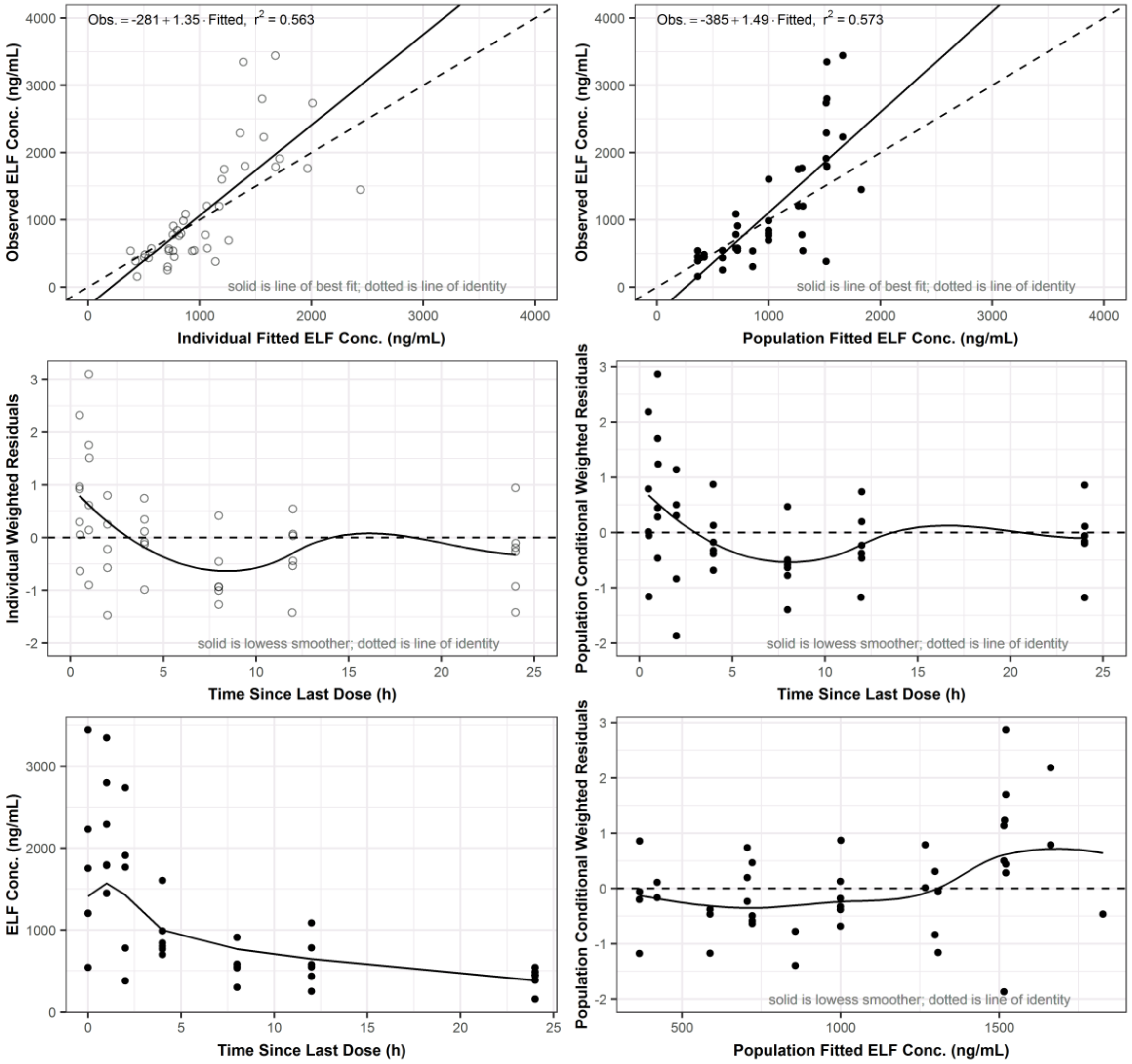

REFERENCES

1. Berg JK, Tzanis E, Garrity-Ryan L, Bai S, Chitra S, Manley A, Villano S. 2018. Pharmacokinetics and safety of omadacycline in subjects with impaired renal function. Antimicrob Agents Chemother 62:e02057-17. https://doi.org/10.1128/AAC.02057-17.
2. Gotfried MH, Horn K, Garrity-Ryan L, Villano S, Tzanis E, Chitra S, Manley A, Tanaka SK, Rodvold KA. 2017. Comparison of omadacycline and tigecycline pharmacokinetics in the plasma, epithelial lining fluid, and alveolar cells of healthy adult subjects. Antimicrob Agents Chemother 61:e01135-17. https://doi.org/10.1128/AAC.01135-17.
3. Bundrant LA, Tzanis E, Garrity-Ryan L, Bai S, Chitra S, Manley A, Villano S. 2018. Safety and pharmacokinetics of the aminomethylcycline antibiotic omadacycline administered to healthy subjects in oral multiple dose regimens. Antimicrob Agents Chemother 62e:01487-17. https://doi.org/10.1128/AAC.01487-17.
4. Overcash JS, Bhiwandi P, Garrity-Ryan L, Steenbergen J, Bai S, Chitra S, Manley A, Tzanis E. 2019. Pharmacokinetics, safety, and clinical outcomes of omadacycline in women with cystitis: Results from a Phase 1b study. Antimicrob Agents Chemother 63:e02083-18. https://doi.org/10.1128/AAC.02083-18.
5. Stets R, Popescu M, Gonong JR, Mitha I, Nseir W, Medej A, Kirsch C, Das AF, Garrity-Ryan L, Steenbergen JN, Manley A, Eckburg PB, Tzanis E, McGovern PC, Loh E. 2019. Omadacycline for community-acquired bacterial pneumonia. N Engl J Med 380:517-27. DOI:10.1056/NEJMoa1800201

6. Noel GJ, Draper M, Hait H, Tanaka SK. 2012. Safety and efficacy of PTK0796 (omadacycline) as treatment of complicated skin and soft tissue infection (cSSTI). Abstr Europ Congr Clin Microbiol Infect Dis, London, United Kingdom.
7. O'Riordan W, Green S, Overcash JS, Puljiz I, Metallidis S, Gardovskis J, Garrity-Ryan L, Das AF, Tzanis E, Eckburg PB, Manley A, Villano SA, Steenbergen JN, Loh E. 2019. Omadacycline for acute bacterial skin and skin-structure infections. N Engl J Med 380:528-38. DOI:10.1056/NEJMoa1800170
8. O'Riordan W, Cardenas C, Shin E, Sirbu A, Garrity-Ryan L, Das AF, Eckburg PB, Manley A, Steenbergen JN, Tzanis E, McGovern PC, Loh E. 2019. Once-daily oral omadacycline versus twice-daily oral linezolid for acute bacterial skin and skin structure infections (OASIS-2): a phase 3, double-blind, multicentre, randomised, controlled, non-inferiority trial. Lancet Infect Dis. Published online August 29, 2019. http://dx.doi.org/10.1016/S1473-3099(19)30275-0.
